# Antifungal Activity and Nail Permeation of Nail Lacquer Containing *Piper regnellii* (Miq.) C. CD. var. *pallescens* (C. DC.) Yunck (Piperaceae) Leave Extracts and Derivatives

**DOI:** 10.3390/molecules15063920

**Published:** 2010-06-01

**Authors:** Andrea Mayumi Koroishi, Elizandra Sehn, Mauro Luciano Baesso, Tânia Ueda-Nakamura, Celso Vataru Nakamura, Diógenes Aparício Garcia Cortez, Benedito Prado Dias Filho

**Affiliations:** 1 Programa de Pós-graduação em Ciências Farmacêuticas, Universidade Estadual de Maringá, Brazil; E-Mail: mayumikoroishi@gmail.com (A.M.K.); tunakamura@uem.br (T.U.N.); cvnakamura@uem.br (C.V.N); dagcortez@uem.br (D.A.G.C.); 2 Pós-graduação em Física, Departamento de Física, Universidade Estadual de Maringá, Brazil; E-Mail: elizandrasehn@hotmail.com (E.S.); mlbaesso@uem.br (M.L.B.); 3 Departamento de Ciências Básicas da Saúde, Universidade Estadual de Maringá, Av. Colombo, 5790, 87020-900, Maringá, Brazil; 4 Departamento de Farmácia e Farmacologia, Universidade Estadual de Maringá, Brazil

**Keywords:** *Piper regnellii*, nail lacquer, dermatophyte, *Trichophyton rubrum*, onychomycosis

## Abstract

The dermatophytes are filamentous fungi that cause cutaneous fungal infections because they use keratin as a nutrient source. For this study the antidermatophyte activity of the extracts and derivates from leaves of *Piper regnellii* was analyzed. From the dichloromethane extract (EBD) neolignans such as eupomatenoid-3 and eupomatenoid-5 were obtained, and it was submitted to fractionation to remove the green residue, designated as the chloroform fraction (FF). Extracts, chloroform fraction and compounds were tested against *Trichophyton rubrum* ATCC 28189 to determine the minimum inhibitory concentration (MIC). The chloroform fraction was incorporated to nail lacquer that was analyzed by photoacoustic spectroscopy, *In vitro* assay and scanning electronic microscopy. For antifungal activity in solid medium the dichloromethane extract and chloroform fraction were used. The compounds eupomatenoid-3 and eupomatenoid-5 were less active than the dichloromethane extract against *T. rubrum.* EBD and FF showed moderate activity in hyphal growth inhibition in solid medium and EBD did not link to ergosterol. Nail lacquer containing the chloroform fraction showed good penetration through the nail as determined by photoacoustic spectroscopy. From *In vitro* studies it was observed that nail lacquer concentrations above 20 mg/mL prevented the growth of fungi, but concentrations up to 2.5 inhibited the growth. Scanning electronic microscopy was used to confirm the *In vitro* nail lacquer activity results. The specie *P. regnellii* showed great antifungal activity against *T. rubrum*, and nail lacquer containing its chloroform fraction has great potential to treat onychomycosis caused by these microorganisms.

## 1. Introduction 

In tropical and subtropical countries, fungal infections are one of the most common skin diseases. Among them, in Brazil, one of the main superficial fungal infections are dermatophytoses. Among the fungal agents responsible for this are the dermatophytes which infect keratinized structures, including nails, hair and skin because they can use the keratin as nutrient source. The genera causing these infections are *Epidermophyton*, *Microsporum* and *Trichophyton* [1]. In the last 30 years, there has been has increased concern about fungal infections due to the increasing the number of immuno-compromised patients and individuals who receive chemotherapy treatment like cancer patients and transplant recipients, treated with immunosuppressive drugs [2]. There is a major concern with the effectiveness of antifungals, due to their limitations and also the toxicity of the same, which can lead to the non-recurrence of the fungistatic effect or lead to resistance over the treatment period [3]. Because of this recently chemical and pharmacological studies on substances derived from plants with therapeutic properties are increasingly taking place.

The family Piperaceae, consisting of the genera *Piper*, *Peperomia*, *Ottonia* and *Pothomorphe*, has medicinal properties widely employed by the population [4]. Regarding the genus *Piper*, phytochemical searches worldwide have isolated many classes of bioactive compounds like alkaloids, amides, propenylphenols, lignans, neolignans, terpens, chalcones, flavones and many others [5]. This genus displays a variety of biological activities, for example the antifungal activity of *Piper arboreum* and *Piper tuberculatum* against *Cladosporium cladosporioides* [6], *Piper fulvescens* against *Trichophyton mentagrophytes* and *Microsporum gypseum* [7,8], moreover, the specie *Piper regnellii* showed activity against *Staphylococcus aureus* and *Bacillus subtilis*, yeasts *Candida krusei* and *Candida albicans* [9], dermtophyte *Trichophyton rubrum*, *T. mentagrophytes*, *Microsporum canis*, *M. gypseum* [10], protozoa like *Leishmania amazonensis* [11] and *Trypanossoma cruzi* [12].

The human nail is composed by highly compressed and keratinized dead cells. Many nail disorders may occur such as dystrophy, hypertrophy, inflammation, infection, *etc* [13]. Onychomycosis is a type of ungual disorder. The main fungi that cause this pathology are *Epidermophyton floccosum*, *T. mentagrophytes* and *T. rubrum* [14].

Oral therapy displays some disadvantages such as systemic adverse effects and drug interactions. To work around this, topical therapy is advantageous because the drug acts directly at the site of action and this possibly reduces the side effects, drug interactions and costs of treatment [15]. The use of nail lacquer formulations containing an antimycotic may act as a regular transungual delivery system thereby facilitating the penetration of the active principles into the nail [15,16].

The aim of this study was to determine the antifungal activity against *T. rubrum* of extract, fractions and compounds from leaves of *P. regnellii*. In addition, a nail lacquer containing the chloroform fraction to treat onychomycosis was developed and the activity of this formulation was analyzed by photoacoustic spectroscopy, *In vitro* assay and scanning electronic microscopy.

## 2. Results and Discussion

A 9:1 hydroethanolic extract, a dichloromethane extract (EBD) and an aqueous extract were obtained from the leaves of plant *P. regnelli.* The EBD was clarified using chloroform as mobile phase, and then called the chloroform fraction (FF). In addition, compounds as eupomatenoid-3 and eupomatenoid-5 were isolated from the EBD. All the extracts and derivates were submitted to the antifungal activity assay. 

The minimal inhibitory concentration of the extract was 15.6 µg/mL, as described by Koroishi et al. [10] and that of the chloroform fraction was 7.8 µg/mL. The two isolated neolignans, eupomatenoid-3 and eupomatenoid-5; showed activity greater than 100 µg/mL and 25 µg/mL, respectively. Nystatin, ketoconazole, fluconazole and amphotericin B were used as standard drugs, and their MIC values were 0.78, 0.39, 3.2 and 0.39 µg/mL, respectively (Table 1). The minimal concentration values required for inhibition of spore germination are also presented in Table 1. The results were satisfactory. The MIC values of the extract and fractions were not significantly different, showing that even after removal of the dark green residue using chloroform in the clarification process the activity is retained. In previous studies different hydroethanolic extracts of the same plant were tested against yeasts, dermatophytes and non-dermatophytes, and the microorganisms most sensitive to the extracts were dermatophytes, *T. rubrum*, *T. mentagrophytes*, *M. canis* and *M. gypseum* [10]. Other *Piper* species contain compounds with antifungal activity, such as the amides of *Piper arboreum* and *Piper tuberculatum*, active against *Cladosporium sphaerospermum* and *C. cladosporioides*, respectively [6]. Danelutte [17] examined the activity of flavonones and prenylated hydroquinones of *Piper crassinervium* against the same fungi, which varied between 1 and 5, and 1 to 10 µg/mL, Freixa et al. [8] studied the activity of three neolignans of *Piper fulvescens*, eupomatenoid-5, eupomatenoid-6 and conocarpan, and only the first one was not active against the dermatophyte *T. mentagrophytes*, while the other compounds showed activity at 1 and 8 µg/mL, respectively. Many other species have been studied for antidermatophyte activity, like species of the family Asteraceae,. For example, the chloroform extract of *Pterocaulon polystachyum* showed better activity at a concentration of 12.5 µg/mL against *T. mentagrophytes* [2]. Prasad et al. [18] checked the activity of 4′-methoxyflavone, a compound isolated of *Psoralea coryfolia* (Fabaceae), with a MIC value of 62.5 µg/mL *vs. T. mentagrophytes* and *T. rubrum* and 125 µg/mL against *E. floccosum* and *M. gypseum*. Among other species *Ziziphus joazeiro* Mart. (Rhamanaceae) and *Caesalpinia pyramidalis* Tul are active against *Candida guilliermondii* and *T. rubrum* with MIC values between 6.25 and 25 µg/mL. In previous studies it was verified that the extracts obtained of leaves from *P. regnellii* significantly inhibited the germination of *T. rubrum* spores at concentration of 7.8.

**Figure 1:**
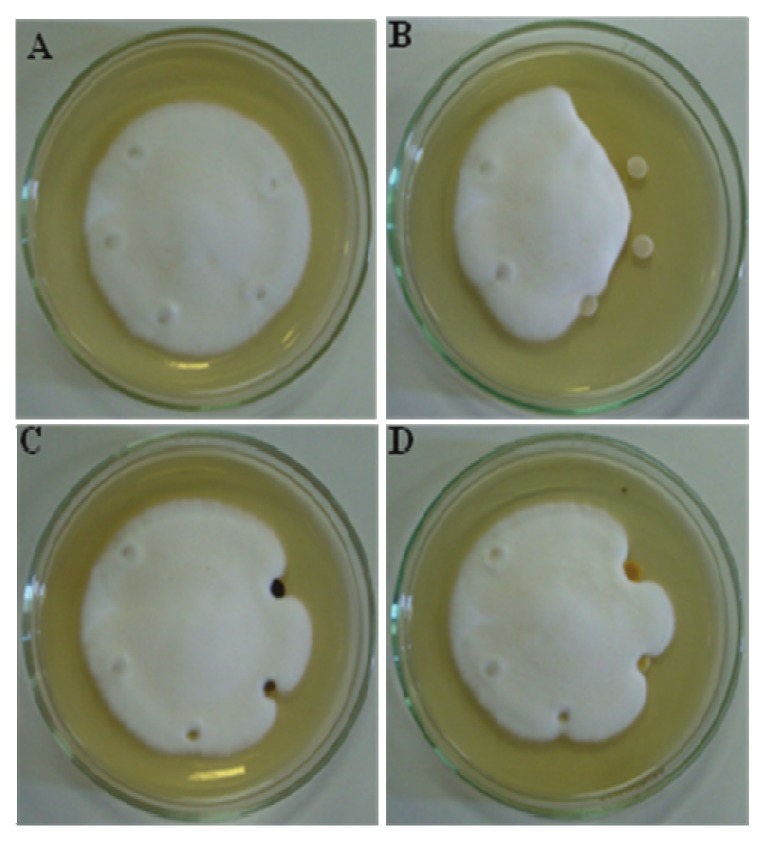
Antifungal active in solid medium against *T. rubrum*. A = Negative control – DMSO 100, 10, 1, 0.1%; B = Amphotericin B – 100, 10, 1, 0.1 µg/mL and water. C = Dichloromethane extract (EBD) and D = Chloroform fraction (FF) – 1000, 100, 10 and 1 µg/mL. The water was used as control, and the concentrations decrease in clockwise fashion.

There are two phases of fungal growth, spore germination and hyphal growth, where drug action can occur. The hyphal growth inhibition was analyzed and the extract and fraction showed moderate inhibition at a concentration of 100 µg/mL, while amphotericin B inhibited it at 10 µg/mL, as observed in the Figure 1. DMSO and water were employed as controls.

**Figure 2:**
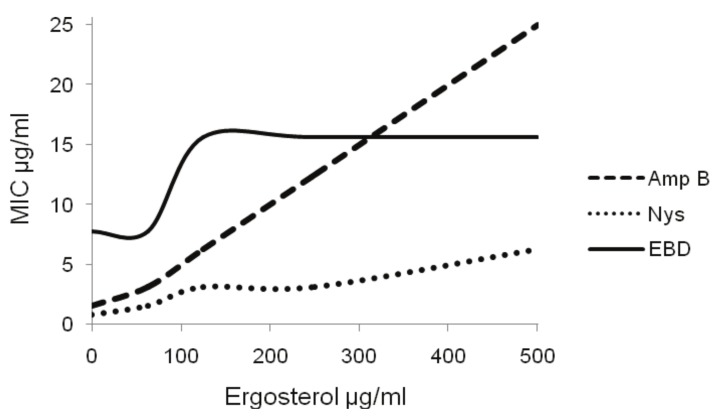
Ergosterol effect assay in *T. rubrum*–Exogen ergoterol (62.5–500 µg/mL) was added on the MIC of the dichlomethane extract (EBD), amphotericin B (Amp B) and nystatin (Nys).

Many different modes of action can be studied, for example, the biosynthesis of ergosterol or the interference of drug-ergosterol binding with cell functions. Ergosterol is lipid that forms part of the membranes of fungi and protozoa. For example amphotericin B binds to ergosterol perturbing membrane functions and causing leakage of cell contents [19]. Figure 2 shows the effect of exogenous ergosterol on the MIC of the drugs.

When the concentrations of ergosterol increase the values of the MIC of amphotericin B also increase. In the absence of ergosterol the MIC of this drug was 1.56 µg/mL, and by varying the substrate between 0 to 500 µg/mL, the MIC changed from 1.56 to 25 µg/mL, indicating binding with the ergosterol. Despite this, the extract and nystatin did not have the same activity as amphotericin B, because when the ergosterol concentration increased the MIC values remained the same.

**Figure 3:**
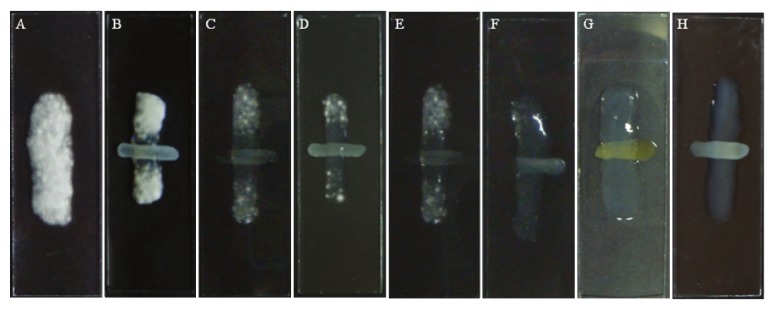
Antifungal activity of nail lacquer with different concentrations of extract against *T. rubrum*. A = Control; B = Nail lacquer without fraction; Nail lacquer with fraction: C = 2.5 mg/mL; D = 5 mg/mL; E = 10 mg/mL; F = 20 mg/mL; G = 40 mg/mL; H = Micolamin.

**Figure 4:**
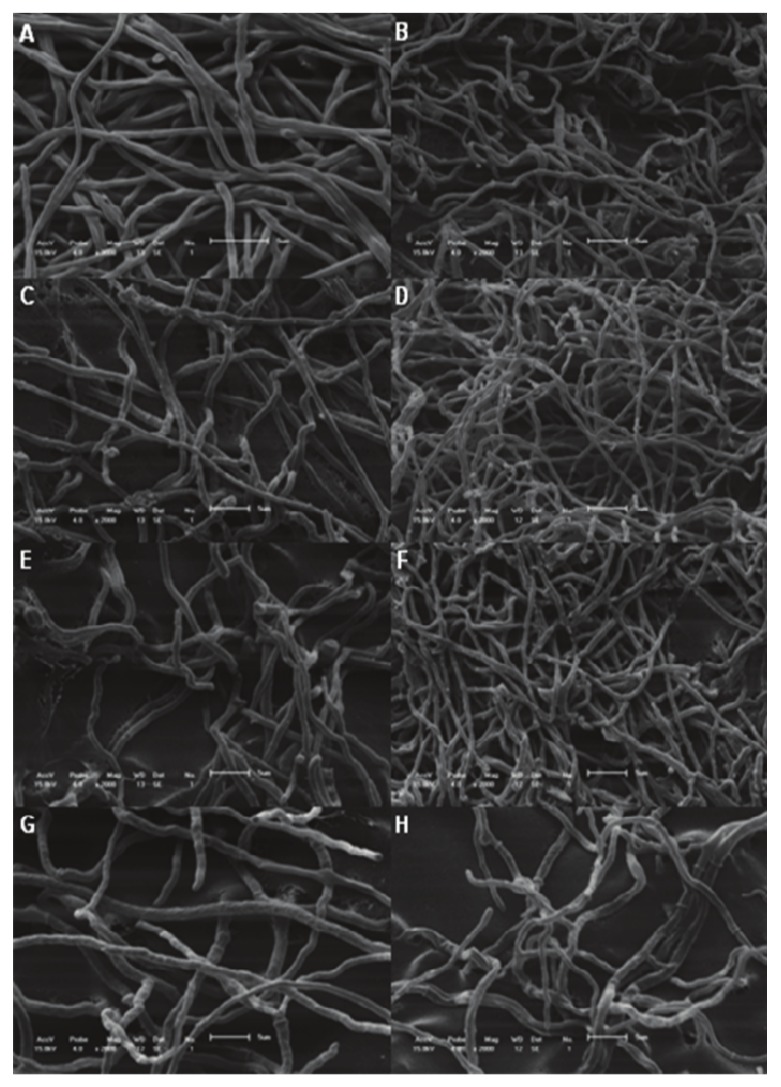
Scanning electronic microscopy of *Trichophyton rubrum* treated with different concentrations of fraction in nail laquer. A and B = control; C and D = nail lacquer without fraction; E and F = nail lacquer with 1.25 mg of chloroform fraction; G and H= nail lacquer with 5 mg of chloroform fraction. C, E and G= samples taken near the nail lacquer; D, F and H=samples taken far the nail lacquer. Magnification: 3000× (A) and 2000× (B–H). Bars 5 µm.

The chloroform fraction was incorporated into nail lacquer at various concentrations ranging between 0.3 to 40 mg per mL. The concentration of 20 mg/mL did not present visible growth compared the control (Figures 3F and 3A). However, concentrations above 2.5 mg/mL inhibited the growth of fungi (Figure 3C) at the point of application of the nail lacquer. Figure 4 shows images of MEV of *T. rubrum*. As previously mentioned, in this method was possible to confirm the reduction of the growth of cultures treated with nail lacquer containing chloroform fraction. There is a reduced growth of cells treated near the nail lacquer (Figures 4C, 4E and 4F). Comparing the Figures 4D, 4F, 4G could see cell growth was lower at a concentration of 5 mg/mL. It is possible the active compounds spread by the agar and in higher concentrations the compounds inhibited the growth.

**Figure 5:**
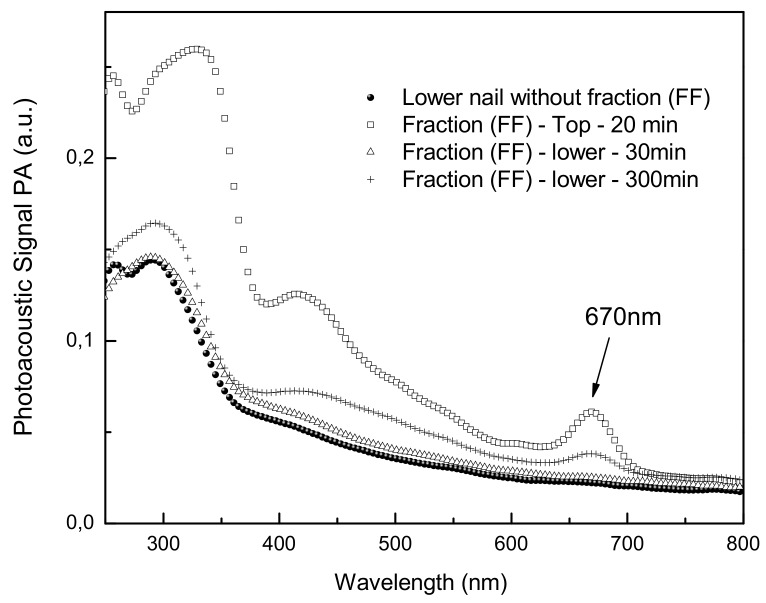
Photoacoustic Spectroscopy Measurement. after 20 min the FF fraction was detected at 670 nm. Spectra obtained with excitation performed in the nail. The light modulation frequency was 20 Hz. The inset highlights the presence of the FF fraction absorption in the spectrum. With this procedure the penetration depth in the nail (the thermal diffusion length) corresponds to about 670 nm.

The human nail is susceptible to a large number of fungal (onychomycosis) and other infections [21]. The photoacoustic spectroscopy technique if used to measure the thermal diffusivity (α) of human nails [21]. Figure 5 shows the spectra of the drugs on the nail. The medicine was placed on top of the nail and a top spectrum was obtained soon after. The graphic shows the curve of the absorption spectrum of the bottom of the nail 30 min after application of FF fraction. After 5 h of application of the product, could be performing the spectrum observed in the inner part of the permeation of the nail. The band around 670 nm becomes visible, showing the permeation. The nail lacquer containing the FF fraction showed good penetration in the nail and the absorption is still equal after extending the time. Nuglisch et al. [22] verified that using two formulations contained cyclopirox the propagation rate through the nails in different samples from healthy volunteers showed that the photoacoustic spectroscopy technique is a useful tool to analyze the penetration of topical formulations in human nails.

## 3. Experimental

### 3.1. Plant Material

The leaves from *Piper regnellii* (Miq.) C. CD. var. *pallescens* (C. DC.) Yunck (Piperaceae) were collected in April 2006 in Horto of Medicinal Plants “Profª. Irenice Silva” on the Campus of Universidade Estadual de Maringá. The plant material was identified by Marilia Borgo of the Botanical Department of Universidade Federal do Paraná, and a voucher specimen (HUM 8392) is deposited at the Herbarium of Universidade Estadual de Maringá, Paraná, Brazil.

### 3.2. Preparation of Plant Extract and Fractions

Dried and powdered plant material (leaves) was extracted by maceration with 9:1 ethanol-water at room temperature at a leaves:solvent ratio of 1:10 (w/v). After the extract was filtered and the filtrate was evaporated to dryness under reduced pressure at 40 °C to give an aqueous extract and a dark green residue which was washed with dicloromethane and the organic solvent removed to give the dicloromethane extract that was submitted to two processes. First, to isolate the compounds eupomatenoid-3 and eupomatenoid-5 the techniques already described in previous studies [10,12,20] were used. The extract was submitted to vacuum chromatography on silica gel eluted with solvents of increasing polarity. The hexane fraction, obtained by this process, was rechromatographed by column chromatography on silica gel 60 (70–230 mesh) eluted with hexane and hexane-chloroform in the following proportions: 49:1, 19:1, 9:1 and 1:1, v/v, chloroform, ethyl acetate, acetone and methanol. The second process, to remove (clarify) the green residue was used *D*-chloroform colunm eluated with chloroform, obtaining the chloroform fraction.

### 3.3. Nail Lacquer Formulation

The formulation used consisted of a solvent volatile (ethanol, ethyl acetate, methanol), apolar soluble polymer (methacrylic acid copolymer, vinyl polymers), plasticizer (triacetine, dibutyl vitalato) and the chloroform fraction, at concentrations between 0.6 to 40 mg/mL. 

### 3.4. Microorganism Used and Growth Conditions

The test specie used for this investigation was *T. rubrun* ATCC 28189. The fungi was maintained on Sabouraud dextrose agar (SDA) slants at 28 °C and subcultured monthly throughout this study.

### 3.5. Antifungal Activity Assay

#### 3.5.1. Microbroth Dilution Assay

Culture was grown on Sabouraud dextrose agar (SDA, Difco Laboratories, Detroit, MI, USA) tubes for 7–14 days, after which time spores were harvested from sporulating colonies and suspended in sterile ion solution. The concentration of spores was adjusted to 1.0 × 10^5^ spores/mL using a hemocytometer. The antifungal assay was performed by the microdilution technique in sterile flat botton microplates. Each well contained appropriate test samples, Sabouraud dextrose broth and approximately 2 × 10^3^–3 × 10^3^ spores in a total volume of 100 µL. The plates were incubated at 28 °C for 72 h. Two susceptibility endpoints were recorded for each isolate. The MIC was defined as the lowest concentration of compounds at which the microorganism tested did not demonstrate visible growth. For this experiment the dichloromethane extract, the chloroform fraction, the isolated compounds and nystatin, amphotericin, fluconazole and ketoconazole at concentrations between 0.2 to 100 µg/mL as standard drugs were used. For comparative purposes, the plates were incubated at 28 °C for 20–30 h and then examined for spore germination under an inverted microscope. For quantification, spores were considered germinated if they had a germ tube at least twice the length of the spore.

#### 3.5.2. Antifungal Activity in Solid Medium

Petri-plates containing 20 mL de SDA medium were seeded with 10 µL of standard spore suspension (10^5^ spores/mL). The plates were incubated in a humidified chamber at a temperature of 28 °C until the diameter growth mycelial growth 2–3 cm. After, the disks were placed at a distance of 0.5 cm from the edge of the colony. About 10 µL of serial dilutions of dichloromethane extract, chloroform fraction, DMSO, and water were added to the disks. The plates were incubated at a temperature of 28 °C until final growth. The inhibition of hyphal elongation was observed by the lack of growth around the disk containing the drug test [18].

#### 3.5.3. Ergosterol Effect Assay

For this assay, the ergosterol was added the Sabouraud dextrose broth medium at four concentrations: 500, 250, 125 and 62.5 µg/mL. The dichloromethane extract was transferred to the first well, a two fold serial dilution was perfomed and the medium was incubated at 28 °C for 72 h. Amphotericin B and nystatin were used as controls [3].

#### 3.5.4. *In vitro* Test with the Nail Lacquer Containing Fraction

In this test, the nail lacquer was applied at glass slides forming a line. Concentrations between 0.3 to 40 mg/mL were analyzed, and Micolamin was used as control drug. The inoculum used had 3 × 10^4^ spores/mL. The microculture was placed in a Petri dish in a moist chamber at a temperature of 28 °C for 5 days. Later, the cultures underwent scanning electronic microscopy. Three portions were removed, far, near and upon the application point.

#### 3.5.5. Scanning Electronic Microscopy

The microculture was fixed in glutaraldehyde 2.5% in 0.1 M cacodilato buffer for 1 day. Later, it was washed in 0.1 M cacodilato buffer. The samples were placed on a specimen support with poly-L-lysine. Subsequently, the samples were dehydrated in graded ethanol, 15–100% for 15 min each. Then, they were submitted to critical-point-drying in CO_2_, coated with chromium in a Penning sputter system in a high-vaccum chamber chamber (Gatan-Model 681), coated with gold and observed with a Shimadzu SS-550 Scanning electron microscope field-emission scanning electron microscope.

### 3.6. Photoacoustic Spectroscopy Measurements

The photoacoustic spectroscopy (PAS) measurements were performed using an experimental setup as shown in Figure 5. The monochromatic light was obtained from a 1000 W xenon arc lamp (Oriel Corporation 68820). The monochromator used was also from Oriel Instruments (model 77250). The light beam was modulated with a mechanical chopper (Stanford Research Systems SR540). The photoacoustic cell was homemade projected to have a minimal volume. It was made of aluminum block, machined to hold samples with maximum dimensions of about 5 mm in diameter and 1 mm thick, which allows light to enter through a high transparent quartz window of 6 mm in diameter and 2 mm thick. The micro-phone chamber was 15 mm away and connected to the sample holder chamber by means of a 1 mm diameter duct. The used capacitive microphone is a very sensitive 12 mm diameter Bruel & Kjaer model 2639, which presents a high gain of 50 mV/Pa and flat frequency response performance from 1 Hz to 10 kHz. The lock-in amplifier was from EG & G Instruments, model 5110. All the photoacoustic spectra were obtained at a modulation frequency of 25 Hz and recorded between 250 and 800 nm. The data acquisition was performed by a personal computer and the PAS spectra were normalized with respect to the carbon black signal. In the photoacoustic measurements, the thermal diffusion length (μ_s_) defines the sample nail [22] depth which contributes to the signal. This parameter is defined as μ_s_= (d/αf)^1/2^, in which d is the sample thermal diffusivity and f the light modulation frequency. With low frequencies one can inspect at long depths beneath the nail surface, while higher frequencies probe the nail surface. This is the well known characteristic of this technique, widely used to perform depth profile analysis. Taking f = 25 Hz and the thermal diffusivity of the nail measured before as d = 8.1 × 10^−4^cm^2^/s [23], the studied samples present thicknesses varying from 400 to 600 μm. The samples were excited first onto the sample external face and after that they were turned upside down to impinge the light in the sample internal face. In this way, the detection of the fraction FF optical absorption bands at the internal side of the sample means that the applied substances propagated through the wound.

## 4. Conclusions

In conclusion, *Piper regnellii* showed effective antifungal activity, although the isolated compounds eupomatenoid-3 and eupomatenoid-5 showed low activity when compared the dichloromethane extract and chloroform fraction. The incorporation of this fraction in a vehicle, nail lacquer, showed good permeation through the nail and good *In vitro* activity against *T. rubrum*. Furthermore, the dichloromethane extract was not able to bind ergosterol.

## Figures and Tables

**Table 1:** Antifungal activity of extract, fraction and isolated compounds of leaves from *P. regnellii* against *T. rubrum*.

	Antifungal Activity	
	Minimal Concentration Required for Inhibition of Spore Germination (µg/mL)	Minimal Inhibitory Concentration (µg/mL)
Dichloromethane extract (EBD)	7.8	15.6
Chloroform fraction (FF)	7.8	7.8
Eupomatenoid-3	>100	>100
Eupomatenoid-5	12.5	25
Amphotericin B	0.2	0.4
Nystatin	0.4	0.8
Fluconazole	1.6	3.2
Ketoconazole	0.2	0.4
